# Lewis Hall Sayre, M. D.

**Published:** 1890-02

**Authors:** 


Lewis Hall Sayre, M. D., of New York, son of the well-known surgeon, Dr. Lewis A. Sayre, died suddenly on the night of January 2, 1890, of heart failure. He returned late at night to his father's residence, No. 285 Fifth avenue, and was found by the family the next morning, sitting in a chair in his office, cold in death. An autopsy showed disease of the liver and kidneys. The *Medical Record* gave the following account of his life:

Dr. Sayre was born in 1851, in New York. He obtained his general education in the College of the City of New York, and his professional diploma from Bellevue Hospital Medical College. He was an assistant professor of orthopedic surgery in the Bellevue Hospital Medical College, a member of the Academy of Medicine, and of several State and National Medical Societies.

The sympathy of all will be extended to Dr. Sayre, who, for the second time, has suddenly lost a son.

